# Shading effect on physiological parameters and in vitro embryo production of tropical adapted Nellore heifers in integrated crop-livestock-forest systems

**DOI:** 10.1007/s11250-020-02244-3

**Published:** 2020-03-06

**Authors:** Wilian Aparecido Leite da Silva, Ralf Poehland, Caroline Carvalho de Oliveira, Mariane Gabriela Cesar Ribeiro Ferreira, Ricardo Garcia de Almeida, Mirela Brochado Souza Cáceres, Gustavo Guerino Macedo, Eliane Vianna da Costa e Silva, Fabiana Villa Alves, Eriklis Nogueira, Fabiana de Andrade Melo-Sterza

**Affiliations:** 1 Animal Science, State University of Mato Grosso do Sul, Aquidauana, Mato Grosso do Sul Brazil; 2 Leibniz Institute for Farm Animal Biology, Institute of Reproductive Biology, Dummerstorf, Germany; 3 Veterinary Sciene, Federal University of Mato Grosso do Sul, Campo Grande, Mato Grosso do Sul Brazil; 4 Animal Science, State University of Londrina, Londrina, Paraná Brazil; 5 Brazilian Agricultural Research Corporation, EMBRAPA Beef Cattle, Campo Grande, Brazil; 6 Brazilian Agricultural Research Corporation, EMBRAPA Pantanal, Corumbá, Brazil

**Keywords:** BGHI, Bovine, Embryo, Heat stress

## Abstract

The aim of this study was to evaluate the impact of increased shadow supply in integrated crop-livestock-forest systems on in vitro embryonic development and physiological parameters related to stress response in Nellore heifers (*Bos indicus*). For the study, animals (*n* = 16) were randomly divided into two groups and kept in areas with different afforestation systems, the integrated crop-livestock-forest (ICLF) and the integrated crop-livestock (ICL) system. The microclimate of the ICLF system provided better comfort conditions than ICL. No differences of respiratory rate, rectal temperature, cortisol, T3, T4, oocyte quality, and cleavage rate between the systems were verified. A higher blastocyst rate was observed in the ICLF (*p* < 0.05). The results demonstrate that Nellore heifers managed in ICLF during summer in Midwest of Brazil showed higher production of in vitro embryos, without typical changes in its physiological parameters. The results observed in the present study indicate that zebu females are able to respond satisfactorily to the intense heat conditions; however, we believe that the long period to which these animals are exposed to these conditions interferes in the oocyte competence and embryo development.

## Introduction

The environment in which animals live has important effects on animal welfare conditions and can affect the productive and reproductive performance of the animals. The satisfactory performance of an animal is dependent on a temperature range called thermal comfort zone (TCZ) which corresponds to temperature limits in which the animal is in homeostasis, without the use of thermoregulatory devices (Pereira 2005). When the ambient temperature reaches critical temperatures higher than TCZ, the efficiency of the heat loss processes is reduced and the animal enters heat stress (HS)(Silanikove 2000), which leads to a consequent activation of thermoregulation mechanisms (Rodrigues et al. 2010). As responses, changes in animal behavior, body temperature, respiratory frequency, heart frequency, and sweating rate (Paranhos da Costa 2004), as well as alterations in adrenal activity and immune response, are observed (Geraldo et al. 2012), Macedo and Zúccari 2012). Reduction of weight gain (Navarini et al. 2009), food consumption (Geraldo et al. 2012), and consequently a reduction on reproductive performance has also been reported (Lima et al. 2013).

In reproduction, HS affects follicular development and oocyte quality (Roth et al. 2001), interfering from the initial stages to the end of maturation (Paula-Lopes et al. 2012), as well as the number and quality of embryos produced in vitro (Torres-Junior et al. 2008). As a consequence of the mentioned disorders, the pregnancy rate is also negatively affected.

Shading decreases the incidence of radiation on the animal; it benefits the thermal comfort and favors the homeothermia (Geraldo et al. 2012). According to Silanikove (2000), well-designed shading reduces the total heat load by 30 to 50%. The benefits of shade are most evident in *Bos taurus*, but positive shading effects have already been observed in *Bos indicus* (Navarini et al. 2009).

Although the positive effect of shading is known, important changes in the management of beef cattle in Brazil have not been observed over the years.

The ICLF system, initially designed for the recovery of degraded soils and pastures, was highlighted by improving feed conversion, weight gain, and microclimate, reducing heat through the trees, and contributing to the sustainability of livestock farming in the tropics with a direct effect on welfare and thermal comfort (Broom et al. 2013).

There is a lack of information on the variation in the behavior and reproductive performance of cattle, especially zebu, in relation to how shade is available or distributed in areas where animals graze, for example, using integrated crop-livestock forest system (ICLF).

The aim of this study was to evaluate the impact of the increase in shade supply in the ICLF on in vitro embryonic development and physiological parameters related to stress response of Nellore donors.

## Materials and methods

The experiment was carried out at the Brazilian Agricultural Research Corporation, EMBRAPA Beef Cattle, located in the municipality of Campo Grande, MS (20° 27’ S, 54° 37’ W, and 530 m altitude), in the transition between humid mesothermic climate without drought and tropical humid, with rainy season in the summer and dry in the winter. The rainy season usually occurs between October and March. Annual rainfall is on average 1225 mm (Gemtec 2016).

### Experimental areas

**Fig. 1:**
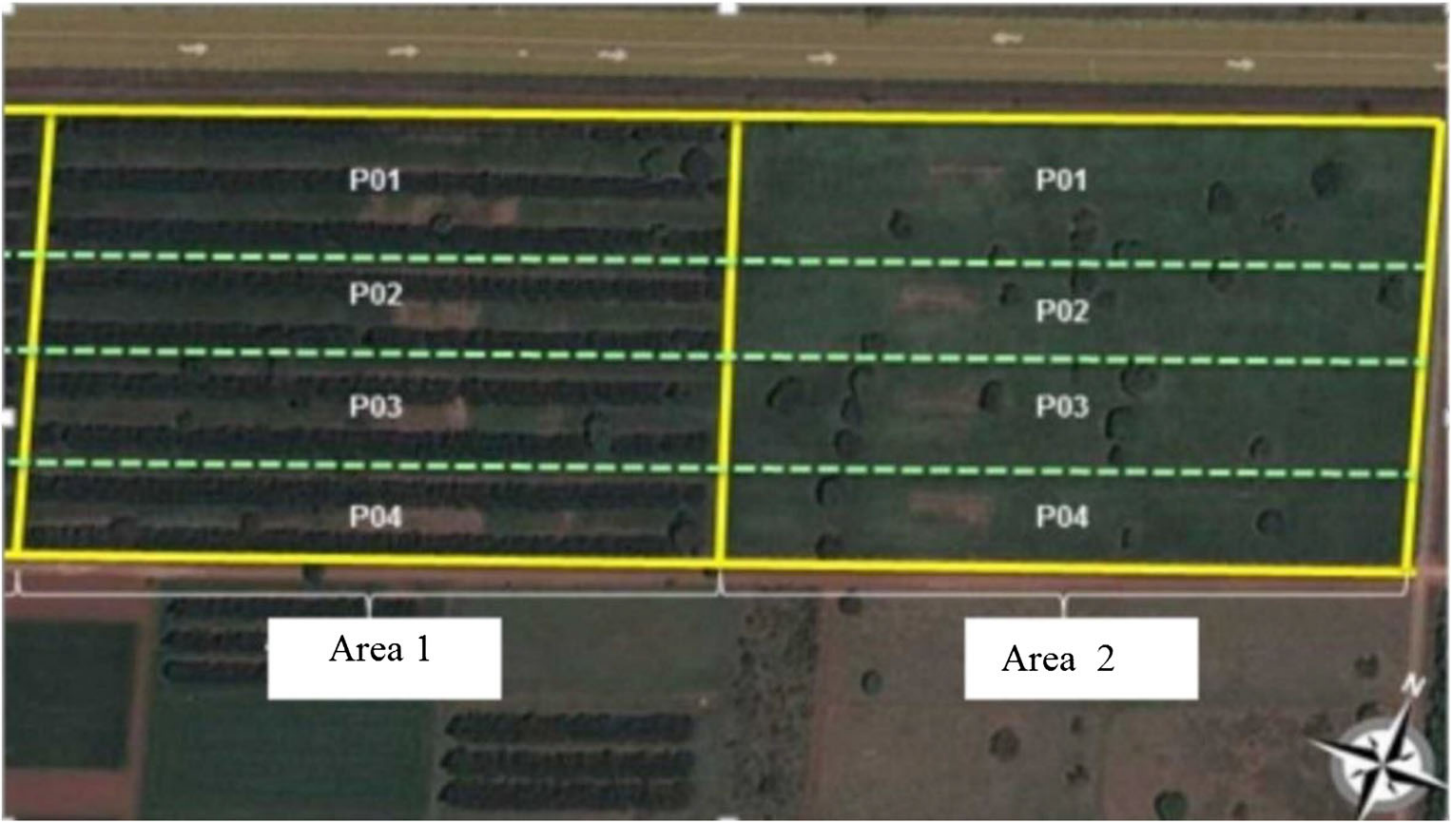
Experimental areas: area 1 ICLF and area 2 ICL. ICLF presented 227 trees ha^−1^ arranged in rows of eucalyptus with 22 m between rows. ICL presented 5 native trees ha^−1^ (Cambará and Cumbaru). https://www.google.com.br/maps/@-20.4139254,-54.708154,606 m/data =!3 m1!1e3

The experimental design was completely randomized. Two experimental areas were used, like previously described by Oliveira et al. (2014) (Fig. 1). Area 1 was characterized by the integrated crop-livestock-forest system (ICLF) with 6 ha divided into 4 paddocks, with a density of 227 trees ha^−1^ arranged in rows of eucalyptus trees (*Eucalyptus grandis x urophylla*, clone H13) with a distance of 22 m between rows. Area 2 was characterized by pasture with native afforestation of integrated crop-livestock (ICL) with 6 ha divided into 4 paddocks and presence of 5 trees ha^−1^. Native trees from Brazilian Savanah, Cambará (*Gochnatia polymorpha Less*.), and Cumbaru (*Dipteryx alata Vog*) were present. Both areas had *Brachiaria brizantha* cv. Piatã.

The amount of shade in each area was calculated according to the methodology used by Silva (2006) and Cipriani et al. (2015). The height of the tree, stem height, crown radius, and crown type (lentiform, cylindrical, conical, or elioid) were measured, and from these measurements, the shadow projections of each tree were estimated in each area.

### Animals

Nellore females (*n* = 16) were divided into 2 groups, eight animals per experimental area. The animals were between 22 and 24 months age and had a body score of 2.5–3.5 (Ferguson et al. 1994), with average weight of 348.5(± 41.9). The grazing system used was continuous. The animals had access to water and mineral salt ad libitum. All donors underwent three sessions of ultrasound-guided follicular aspiration (OPU) to collect cumulus-oocyte complexes (COCs) once a month for in vitro embryo production from December 2015 to February 2016.

The heifers were managed in the same areas since weaning.

### Microclimatic monitoring

**Fig. 2:**
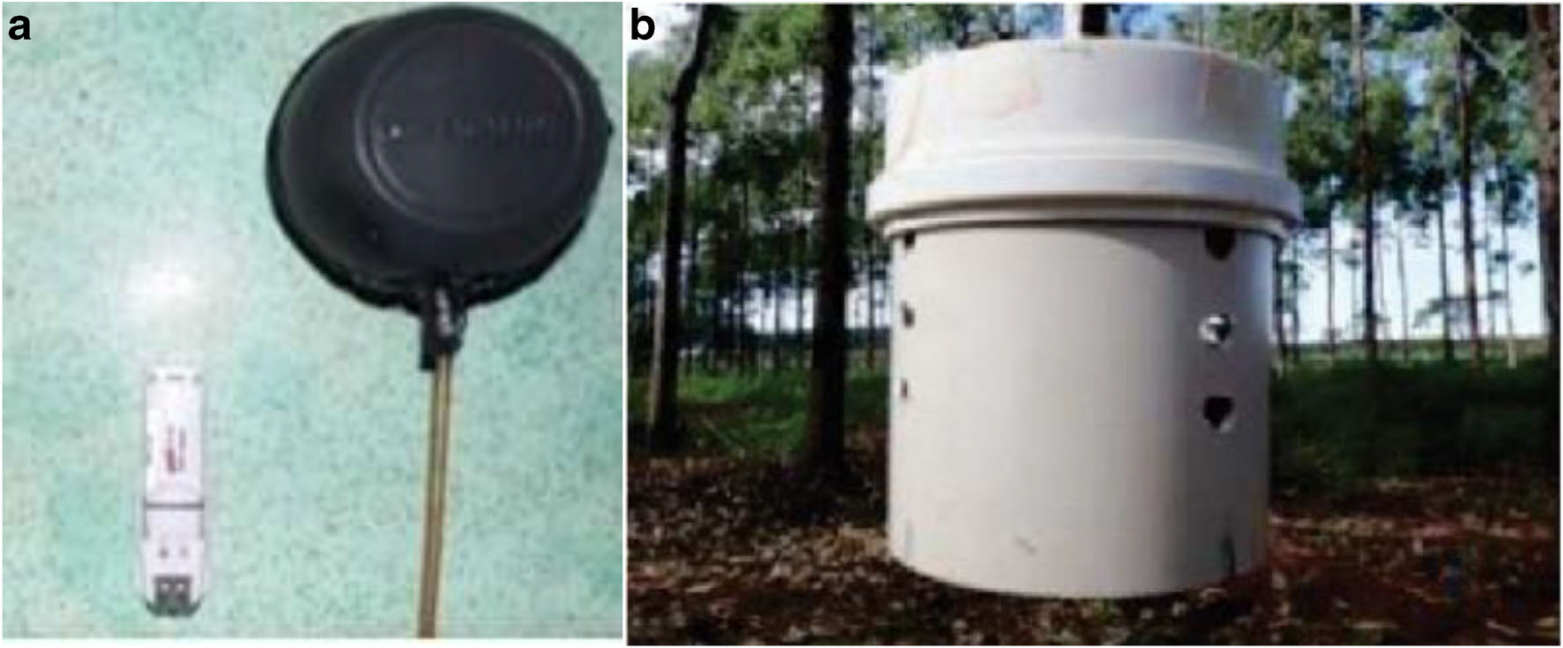
Component devices of the micrometeorological station. **a** Digital thermo-hygrometers with data logger, black globe. **b** Perforated micrometeorological shelter next to the data logger

The microclimatic characteristics in both areas were determined in December 2015, January, and February 2016, from 8:00 a.m. to 4:00 p.m. (local time, GMT 04:00), divided into 8–10, 11–13, and 14–16 h, 4 days per month according to the methodology of Karvatte et al. (2016). For the measurement of black globe temperature (°C), the methodology proposed by Coelho et al. (2013) was adopted, using digital thermo-hygrometers with data logger (model Perceptec1020, Perceptec Soluções e Tecnologia Ltda., São Paulo, Brazil) inserted in plastic buoys (PVC) of 0.15 m in diameter, painted matte black (Fig. 2a).

For the determination of the temperature (°C) and humidity of the air (%), digital thermometer with data loggers (brand Instrutherm, model HT-500) was used to measure dry bulb and dew point (Fig. 2a), inserted in perforated PVC pipes (Fig. 2b) (Trumbo et al. 2012).

The microclimate conditions were accompanied by two devices located in each area.

The devices were placed in full sun and under the projected shade, two meters from the trunk of the trees, 1.3 m above the ground (in an attempt to simulate the height of the back of a young bovine).

The black globe-humidity index (BGHI) was calculated in accord with Buffington et al. (1981): BGHI = Tgn + 0.36.tpo + 41.5, where Tgn = black globe temperature in °C and Tpo = dew-point temperature in °C.

### Collections and evaluations

Heifer BW was measured every 30 days, after a 12-h period of water and feed withdrawal.

For IVP, all of the reagents used for media preparation were from Sigma®.

For reduction of peristaltic movements and less discomfort of the animal, 5 ml of a combination of 2% lidocaine (Person®) and 1% acepromazine (Vetnil®) in the epidural space was injected. For the aspiration of the follicles, an ultrasound (Aquila PRO®, Pie Medical, coupled to the 8 MHz microconvex transducer) connected to a silicone hose with 2 mm of internal diameter, and 80 cm of length and disposable hypodermic needles 20G (50 mm × 9 mm; Terumo®, Bio Brazil) was used. The vacuum pressure was 80 mmHg, maintained with a suction pump (BV-003D WTA, Brazil). The aspiration medium consisted of Dulbecco’s Phosphate Buffered Saline (DPBS) plus 20,000 IU/L of sodium heparin, maintained at 30 °C during aspiration. The recovered COCs were screened and classified immediately after the OPU session. COCs with one or more layers of compact cumulus cells and homogeneous cytoplasm were considered of good quality.

The aspirated COCs were taken to the laboratory in maturation medium in LABMIX transport incubator (WTA, Brazil). The recovered COCs were classified according to the appearance and distribution of cumulus cells and cytoplasmic uniformity (Stojkovic et al. 2001). The selected COCs of all donors of each group were mixed to minimize donor effect and matured for 24 h in drops of 100 μl of maturation medium (TCM199 with Earls’ salts, fetal bovine serum 5% v/v, 01 mE/ml LH, 200 mM l-glutamin, 0.01 mg/ml streptomycin, 10 U/ml penicillin) under mineral oil. After 24 h of maturation, the COCs were washed (1x) and transferred to fertilization medium (supplemented with heparin, TALP, and BSA 6 mg/ml). Cryopreserved semen of a single Nellore bull was used for in vitro embryo production. Spermatozoa were separated by Percoll method and the inseminating dose was 1 × 10^6^sperm/ml. COCs and sperm were co-incubated for 22 h. After washing, the zygotes were transferred to 100 μl SOF under mineral oil for 7 days (38.5 °C and 5% CO_2_ in air). The cleavage rate was determined on day 3 and blastocyst rate on day 7.

Before OPU sessions, blood samples were collected to analyze serum hormone concentrations (cortisol, thyroid hormones (triiodothyronine/T3 and thyroxine/T4)). They were determined by radioimmunoassay (RIA) in solid phase, in an accredited laboratory, using commercial kits specific (ELISA kit) for each hormone.

The respiratory rate was measured by counting the movements/minute of the right flank of the animals for 15 s, which were multiplied by four. The rectal temperature was measured using a digital veterinary thermometer. The superficial body temperature of the rump region, named as skin temperature, was measured using an infrared thermometer. All these parameters were measured immediately before the OPU.

### Statistical analysis

Statistical analysis was performed by PROC GLIMIX (SAS® 2002). For each experimental group, the analysis was performed considering the fixed effects, group, and treatment, on the reproductive variables: oocyte quality, cleavage rate; hormonal parameters: serum level of cortisol, T3, and T4; and physiological parameters: rectal temperature, respiratory rate, and skin temperature.

The graphics were created using GraphPad Prism version 7®.

## Results

The average initial weight of the animals was 319.38(± 29.2) kg for ICLF and 362(± 14) kg for ICL (*p* > 0.05), and the weight at the end of the experiment was 349(± 30.2) and 376(± 15.21) kg (*p* > 0.05), respectively.

The shadow projection was calculated and ICLF offered 8189.4 m^2^ ha^−1^ and ICL 195.7 m^2^ ha^−1^.

**Fig. 3:**
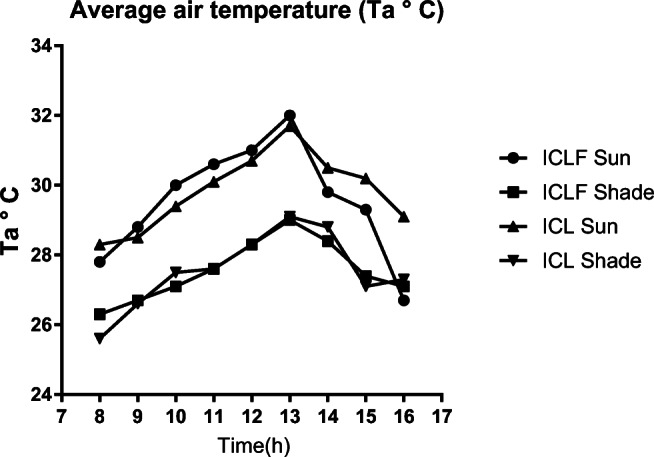
Average air temperature (Ta, °C) recorded in the sun and shade, in integrated crop-livestock-forest and integrated crop-livestock systems (ICLF and ICL) between December and February

**Fig. 4:**
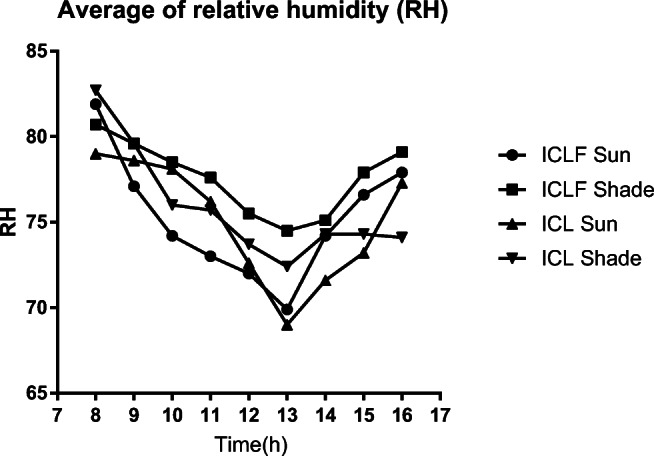
Average of relative humidity (RH), in sun and shade, in integrated crop-livestock-forest and integrated crop-livestock systems (ICLF and ICL) between December and February

The highest temperatures over the 3 months of the experiment (29.4 to 38 °C) were observed between 10 and 14 h in both areas in the sun (Fig. 3). In both groups, the shadow could reduce the temperature by up to 3 °C during the hottest periods. The relative air humidity varied between 72 and 82.7% throughout the experiment (Fig. 4). The pluviometric index recorded in December, January, and February (data collection period) were 139.8 mm, 344.8 mm, and 152 mm respectively. The high humidity of January must be highlighted, which could explain the lower temperature averages.

The microclimatic stations recorded higher humidity in the shade, both in ICL and ICLF. The lowest values of relative air humidity were recorded at 1:00 p.m., in the sun, with rates of 69.9 and 69.5% for ICLF and ICL, respectively.

Table 1 shows the monthly average of the black globe temperature and humidity index (BGHI) in the experimental period. There were differences in BGTHI between treatments (*p* = 0.0349), where ICL presented higher index. January presented lower rates of BGHI, due to the higher rainfall index. February stood out with higher values of BGHI, mainly in the sun, at the hottest time of day (13:00), when the highest BGHI values were recorded throughout the experiment, 89.3 in the ICLF and 90.4 in the ICL. On the other hand, it is emphasized that a single time during the experimental period the BGHI was less than 74, and this occurred under shaded area at 8:00.

**Table 1:** Monthly average of the black globe temperature and humidity index (BGHI), in sun and shade, in integrated crop-livestock-forest and integrated livestock systems (ICLF and ICL) between December and February

Systems	Month	Place	BGHI								
			Time point								
			8	9	10	11	12	13	14	15	16
ILCF	December	Sun	85.4	87.2	87.3	86.1	86.6	88	84.6	80.7	79.8
		Shade	77.2	78.7	78.1	78	79	81.2	81.8	80.9	79.8
	January	Sun	75.5	76.1	78.3	77.7	78.3	79.5	78.4	76.3	75.1
		Shade	75.0	74.5	75.6	76.3	76.4	77.2	77.2	77.8	74.8
	February	Sun	82.7	85.9	88.9	89.3	89.7	89.3	90.2	79.7	79.6
		Shade	76.6	78.3	81.1	82.9	82.8	83	82.2	79.5	77.8
ILC	December	Sun	85.2	85.9	86.1	85.6	86.6	88.2	85.7	81.9	82.4
		Shade	78.3	78.5	78.7	79.6	81	82.2	81.1	77.9	80.7
	January	Sun	74.7	76.3	79.4	79.7	78.8	79.5	79.9	78.2	76.6
		Shade	73.8	75	75.8	75.7	75.9	76.8	75.5	77.7	74.7
	February	Sun	82.5	85	89.1	89.8	89.7	90.4	88.5	82.7	79.9
		Shade	78.7	78.5	80.4	81.4	81.7	82.4	82.9	79.9	77.3

None of the measured physiological parameters were higher than the physiological patterns of the species (Rosenberger 1983) (Table 2). There was no difference among the parameters between shading systems.

**Table 2:** Physiological and hormonal parameters from Nellore heifers managed in integrated crop-livestock-forest and integrated crop-livestock systems (ICLF and ICL) between December and February

Systems	ST (°C)	RF (min-1)	RT (°C)	T3 (nmol/L)	T4 (nmol/L)	Cortisol (nmol/L)
ICLF	30.6 (± 1.48)	36.3 (± 5.5)	39.1 (± 0.6)	5.18 (± 1.4)	107.6 (± 23.8)	78.78( ±46.6)
ICL	31.38 (± 1.48)	37.4 (± 6.8)	39.1 (± 0.6)	5.3 (± 1.4)	73.1 (± 30.6)	74.98 (± 21.0)
*P* value	0.12	0.56	0.70	0.90	0.12	0.88

*ST* skin temperature, *RF* respiratory rate, *RT* rectal temperature, *T3* triiodothyronine, *T4* thyroxine

No statistical difference was observed in the oocyte quality and cleavage rate; however, a higher blastocyst rate (*P* = 0.01) was observed in ICLF (Table 3).

**Table 3:** Oocyte quality and embryo production of Nellore heifers managed in integrated crop-livestock-forest and integrated crop-livestock systems (ICLF and ICL) between December and February

System	Oocyte quality (%)	Cleavage rate (%)	Blastocyst rate (%)
ICLF	68.15 (282/409)	48.07 (78/154)	32.5 (50/154)
ICL	73.8 (245/311)	29.4 (55/169)	17.8 (30/169)
*P* value	0.51	0.09	0.01

## Discussion

It has been reported that shading reduces the radiant heat load on animals. As a consequence, it also reduces body heat and facilitates thermoregulation, favoring animal welfare and productivity (Salla et al. 2009). A 30% reduction in the incidence of thermal load in rotational grazing (Paranhos da Costa 2004) was reported, making the environment more comfortable and favorable to grazing. In the present study, ICLF offered around 8 times more shadow (8189.4 m^2^ ha^−1^) than ICL (195.7 m^2^ ha^−1^), and as expected, shadow was efficient in improving the microclimatic conditions that could be demonstrated by the lower BGHI in ICLF.

The National Weather Service (1976) delimited values of BGHI up to 74 as a comfortable situation for cattle, values above that are considered heat stress. However, the need for adjustments of thermal comfort indexes (BGHI and THI) for zebu and their crossbreed has been discussed in recent years (Fialho et al. 2018). In the present study, the highest values of BGHI were verified between 11:00 and 13:00. Along the experimental period, a single time, the BGHI, was less than 74, and this occurred in the shadow at 8:00. At 13:00 under no conditions were BGHI below 76 and if we disregarded January, due to the high humidity (rainfall higher than normal for this period), these values were not lower than 88 in the sun and 81 in the shade, indicating the intense heat to which these animals were under. Looking for better thermal comfort, a study was conducted some years ago in the same region, comparing ICLF with different eucalyptus density and dispersed native tree systems (Karvatte et al. 2016). The authors verified that eucalyptus with a spacing of 22 m between rows promoted better comfort (BGHI) than systems with dispersed trees and ICLF with a spacing of 14 m between rows (high tree density). These results showed that there is a limit in providing shade. It is possible that the high tree density has provided excessive moisture rise, making the environment uncomfortable. Therefore, we decided to conduct our investigation in ICLF with tree spacing of 22 m between rows. In general, throughout the experiment, the humidity was higher than 69% (± 3.5) that means higher than the superior comfort limit, since environments with relative humidity between 60 and 70% are considered comfortable (Baêta and Souza 2010), but high humidity and high temperatures are expected during summer (rain season) in the studied region, and then, it is important to look for management systems that improve animal welfare and consequently animal production.

Oliveira et al. (2014) showed that ICLF with a higher tree density (357 trees ha^−1^) provides a lower weight gain per hectare of Nellore heifers when compared with systems with lower tree densities, 227 trees ha^−1^ and 5 trees ha^−1^, which had similar weight gain per hectare in summer. These results showed that ICLF with intermediate tree density is a good way for livestock farming providing animal welfare without decreasing animal production per hectare and diversification of their revenue sources.

It is known that mammals subjected to heat stress increase the rectal temperature (Hansen 2004; Titto et al. 2011) and respiratory rate (Medeiros and Vieira 1997), making both parameters eligible to evaluate heat stress in cattle (Ferreira et al. 2006). Respiratory frequency is generally the first physiological parameter increasing in situations of heat stress since it is an important physiological mechanism for heat dissipation (Paranhos da Costa 2004). However, about 70–85% of maximal heat loss occurs via evaporation and is due to sweating with the remainder due to respiration. As air temperatures approach those of skin temperature, evaporation becomes the major route for heat exchange with the environment (Hansen 2004).This situation was observed in our study, since the average air temperature during the experiment was 32 °C, in ICLF and ICL, and skin temperature was 30.6 °C and 31.4 °C (*p* > 0,05), in ICLF and ICL respectively.

Melo-Costa et al. (2018) showed that the energetic costs of the thermoregulation seem to be lower in Nellore animals when compared with other breeds reared in tropical environments. The metabolic heat production, respiratory rate, and cutaneous evaporation were stable in an air temperature range of 24 to 35 °C, similar to this study.

In the present study, it was never observed that the respiratory rate and rectal temperature exceed the reference limit for the species (Vasconcelos and Demetrio 2011). Taking into account, in the experimental current conditions, although BGHIs of up to 90.4 were observed, we cannot confirm that the animals were under heat stress. On the other site, it is important to consider that the physiological measurements were carried out immediately before OPU, and in that time, the heifers were together in the corral under same conditions. Each OPU intercalated heifers of ICLF and ICL and all the procedures (animal gathering until the last OPU) occurred in around 3 h. It is possible that the skin and rectal temperature, as well as respiratory rate, had been adjusted to the corral microclimatic conditions during this time, and therefore, we could not see any difference. It would be more appropriate for the measures to be realized without the need to contain the animals and without disturbing their natural state. However, this possibility is very difficult to perform in cattle, especially for Nellore breed.

Recently, it was showed that Nellore heifers reared in ICLF and ICL have normal and similar rectal temperature; however, the vaginal temperature increased in accord with the BGHI increase and was higher in heifers reared in ICL (Oliveira et al. 2019), showing that despite the clear adaptation to the heat, the heifers responded to changes in the environment conditions.

Alterations in cortisol serum concentrations were very clear after acute stressful situations, like Nellore management on corral (Lima et al. 2017) and after weaning (Chaves et al. 2015). Black Angus heifers assigned to pen with four different shade treatments for 8 weeks during summer showed different serum cortisol concentration in accord with shade treatment (Brown-Brandl 2018). However, Mormede et al. (2007) suggested that hypothalamus-pituitary-adrenal axis (HPA) reactivity in cattle, like ACTH (cortisol) is not a reliable indicator of animal welfare status, due to a lot of controversial results. In addition, the authors discussed that the dynamics in adaptive processes may strongly determine the HPA axis responsiveness of individual cattle at a given time. They believe that this process may involve reversible changes in the density and sensitivity of ACTH and cortisol receptors. In the present study, the heifers born in the farm were evaluated and managed in the same environmental conditions (ICL and ICLF) since weaning that means they were chronic exposed to the microclimatic conditions differentiated by shade amount. The adaptation to these conditions may explain the similar cortisol concentrations between groups.

Thyroid hormones (TH) are considered “markers” of the body temperature. Weitzel et al. (2017) observed a pronounced decline in T3 and T4 levels in dairy cows submitted to heat stress. We expected changes in T3 and T4 concentrations in response to a better thermal comfort in ICLF. However, neither T3 nor T4 altered their physiological level, which is in accord with the similar body temperatures observed in both groups.

In ruminants, studies have confirmed that it takes months for a primordial follicle to reach the preovulatory stage (Britt 2008). It is known that oocytes from preantral follicles are growing and with an intense synthesis of RNA (Oktem and Urman 2010), which makes them sensitive to environmental changes. Torres-Junior et al. (2008) evaluated the effects of heat stress on zebu animals in a climatic chamber and did not observe immediate effects on reproductive functions, such as follicular recruitment or follicular growth pattern, but deleterious effects were observed on the ovarian follicular dynamics and oocyte competence 112 days after heat stress.

Several studies have demonstrated that heat stress negatively affects the blastocyst rate (Al-Katanani et al. 2002; Ferreira et al. 2008, Torres-Júnior et al. 2008). In the present study, the blastocyst rate was 54.7% (*p* = 0.01) higher in the ICLF system. One of the methods of identifying the oocyte competence is the evaluation of its ability to cleave and become blastocyst. Fialho et al. (2018) found that zebu crossbred dairy cows exposed to high THI 60 days prior to OPU session had reduced oocyte quality. In the present study, the heifers were in the same management systems since weaning, ensuring more than an entire process of folliculogenesis under the same microclimatic conditions. We could not confirm heat stress looking for the studied physiological parameters, but it is possible that there are cellular and/or molecular processes related to the heat adaptation going on that we could not access in our study, which could affect embryo development.

On the other hand, whenever the animals grazed the same grass, we could not provide information about the influence of the food quality or feed intake on embryo development. Gamarra et al. (2017) evaluated grass differences and Nellore heifer performance in the same area used in this experiment. The authors showed that in comparison with ICL, ICLF with intermediate tree density (the same of this study) has similar grass and grass leaf dry mass yield in summer, but lower neutral detergent fiber content and higher crude protein content, and similar to our study, no difference in animal weight gain was observed between areas.

It was showed that energy and protein intake can change ovarian IGF system, and that dietary protein concentration can negatively affect oocyte quality (Armstrong et al. 2001). Therefore, it is important to investigate if differences on grass composition can be related to oocyte quality and embryo development in different integrated systems.

The results observed in the present study indicate that zebu females are able to respond satisfactorily to intense heat conditions. The higher percentage of in vitro embryo obtained from donors managed in ICLF could be not explained after the designed experiment. But we believe that: (1) measurement of the physiological parameters without animal containment would show differences between systems; (2) molecular changes related to the adaptation to the high BGHI can occur in follicular fluid and complex-cumulus-oocytes that influence oocyte competence and embryo development; and (3) grass composition needs to be analyzed and compared with hormones and factors involved in the oocyte and embryo development.

## Conclusion

Nellore heifers were able to maintain homeothermia in BGHI up to 90.4, without presenting typical physiological changes, indicating to be a thermotolerant breed.

Nellore heifers managed in integrated crop-livestock-forest (ICLF) during summer in Midwest of Brazil showed higher production of in vitro embryos, and we believe that the long period to which these animals were exposed to these conditions improved oocyte competence and embryo development.
